# Non-Surgical Bleeding and Transurethral Resection of the Prostate (TURP) Syndrome after TURP Surgery: A Case Report and Literature Review

**DOI:** 10.3390/pathophysiology31030027

**Published:** 2024-07-12

**Authors:** Akram M. Eraky, Sidney C. Rubenstein, Adnan Khan, Yasser Mokhtar, Nicole M. Gregorich

**Affiliations:** 1 Medical Education Department, Kansas City University of Medicine and Biosciences, Kansas City, MO 64106, USA; 2 Emergency Medicine, Freeman Health System, Joplin, MO 64804, USA; 3 Urology, Freeman Health System, Joplin, MO 64804, USA; pdok@msn.com; 4 Critical Care Medicine, Freeman Health System, Joplin, MO 64804, USA; akhan3@freemanhealth.com (A.K.); ymmokhtar@freemanhealth.com (Y.M.); 5 Department of Medicine, School of Medicine and Public Health, University of Wisconsin, Madison, WI 53726, USA; ngregorich@wisc.edu

**Keywords:** transurethral resection of the prostate, TURP, urokinase, tPA, tissue plasminogen activator, benign prostatic hyperplasia, BPH, fibrinolysis, thrombolysis, TURP syndrome, hyponatremia, post-TURP bleeding, non-surgical bleeding, shock, post-TURP hypotension, bradycardia, tranexamic acid

## Abstract

Patients undergoing transurethral resection of the prostate (TURP) surgery can develop TURP syndrome and post-TURP bleeding. Post-TURP bleeding can be surgical, from arteries or venous sinuses, or non-surgical, due to coagulopathy preventing clot formation. Non-surgical post-TURP bleeding may be due to high concentrations of urokinase and tissue plasminogen activator (tPA) in the urine that cause fibrinolytic changes and increase bleeding risk. Urine urokinase and tPA may have both local and systemic fibrinolytic effects that may prevent blood clot formation locally at the site of surgery, and cause fibrinolytic changes systemically through leaking into the blood stream. Another post-TURP complication that may happen is TURP syndrome, due to absorption of hypotonic glycine fluid through the prostatic venous plexus. TURP syndrome may present with hyponatremia, bradycardia, and hypotension, which may be preceded by hypertension. In this case report, we had a patient with benign prostatic hyperplasia (BPH) who developed both TURP syndrome and non-surgical post-TURP bleeding. These complications were transient for one day after surgery. The local effect of urine urokinase and tPA explains the non-surgical bleeding after TURP by preventing clot formation and inducing bleeding. Coagulation studies showed fibrinolytic changes that may be explained by urokinase and tPA leakage into the blood stream. In conclusion, non-surgical bleeding after TURP can be explained by the presence of fibrinolytic agents in the urine, including urokinase and tPA. There is a deficiency in existing studies explaining the pathophysiology of the fibrinolytic changes and risk of bleeding after TURP. Herein, we discuss the possible pathophysiology of developing fibrinolytic changes after TURP. More research effort should be directed to explore this area to investigate the appropriate medications to treat and prevent post-TURP bleeding. We suggest monitoring patients’ coagulation profiles and electrolytes after TURP because of the risk of developing severe acute hyponatremia, TURP syndrome, fibrinolytic changes, and non-surgical bleeding. In our review of the literature, we discuss current clinical trials testing the use of an antifibrinolytic agent, Tranexamic acid, locally in the irrigation fluid or systemically to prevent post-TURP bleeding by antagonizing the fibrinolytic activity of urine urokinase and tPA.

## 1. Introduction

Benign prostatic hyperplasia (BPH) is a benign tumor in aging men. There are many factors that can affect prostate volume, including hormonal factors, cell receptors expression and signaling, and growth factors [1]. In contrast to other hyperplasia changes in the other parts of human body, it is known that BPH is not a risk factor of prostate cancer [1,2,3,4]. Transurethral resection of the prostate (TURP) surgery is one of the common surgical treatments of BPH. Possible early complications after TURP include TURP syndrome, post-TURP bleeding, urinary tract infection, urinary retention, and urge incontinence. Late complications include bladder neck contractures and urethral strictures [5,6].

The risk of post-TURP bleeding is one of the potential complications after TURP [1,2,4]. This bleeding can be surgical, due to arterial or venous sinus bleeding, or non-surgical, due to coagulation abnormalities that can prevent clot formation or stability. The term “non-surgical bleeding” has been used in the literature to refer to the bleeding caused by a coagulopathy that induces blood clot degradation or prevents clot formation. Although this bleeding may happen after surgery, it is not caused by direct surgical injury of blood vessels [7,8,9]. Non-surgical post-TURP bleeding may be explained by the activity of urokinase, which is a common fibrinolytic agent in the urine. Urokinase may affect both the clot formation locally and coagulation studies systemically [10,11,12]. TURP syndrome is another complication that can develop after TURP surgery. Patients with TURP syndrome may develop severe acute hyponatremia due to absorption of hypotonic glycine fluid through the prostatic venous plexus and hypotension due to bladder distension-induced vasovagal stimulation [13,14,15].

In this case report, we describe a patient who developed post-TURP fibrinolytic changes, non-surgical bleeding, and TURP syndrome presenting with hypotension and hyponatremia. We highlight the importance of checking coagulation studies and electrolytes after TURP surgery. We also discuss the possible pathophysiology of developing fibrinolytic changes after TURP and post-TURP bleeding risk. In our literature review, we mention clinical trials that tested the use of Tranexamic acid as a prophylactic treatment before or during TURP surgery to decrease post-operative bleeding.

## 2. Case Report

A 68-year-old male patient with past medical history of chronic kidney disease stage IIIa, diabetes mellitus type 2, hypertension, and myeloproliferative disease with primary polycythemia presented with complaints of slow urine stream, nocturia, and increased urinary frequency for two years. The patient sought medical advice, and was diagnosed with severe BPH. He tried medical treatments, such as 5-alpha reductase inhibitor and alpha blocker, without any significant improvement. The patient was offered monopolar TURP. The risks and benefits were discussed, and informed consent was obtained. Preoperative labs showed normal hemoglobin and platelet levels. The patient also did not have any signs or symptoms of platelet dysfunction or hypervisconsity syndrome.

General anesthesia was administered, and the patient was placed in lithotomy position. Cystoscopy demonstrated the prostate to have a very large intravesical median lobe. The lateral lobes were extremely obstructive, and the bladder neck was elevated. Using a 27 French monopolar loop electrode, 50 g of tissue were resected down to surgical capsule and sent for histopathologic examination. A rollerball electrode was used to thoroughly fulgurate the surface of the prostatic fossa, which was wide open. A 24 three-way foley catheter was placed with the balloon inflated with 75 cc. The catheter was placed on traction with a Korean knot. A hypotonic glycine fluid was used for irrigation. The duration of the surgery was approximately one hour and 15 min. The resected specimen consisted of multiple pink-tan rubbery tissue fragments with dimensions of 9.5 × 8.5 × 4.0 cm in loose aggregate and weighed about 50 g. Histopathologic examination shows prostate tissue with glandular hyperplasia and scattered areas of mild to moderate chronic inflammation, which is consistent with the diagnosis of BPH.

During surgery, there was no significant venous sinus or arterial bleeding. There were no apparent intraoperative complications. After waking up and being extubated in the operating room, the urine outflow was clear. In the post-anesthesia care unit (PACU), the patient developed hematuria. The patient’s hematocrit of 39% was satisfactory and not indicative for blood transfusion. A single bag of six units of platelets was given to the patient without significant improvement. At the same time, the patient developed acute hyponatremia of 97 mEq/L that was treated with hypertonic saline followed by normal saline. Ephedrine was administered to keep the mean arterial pressure above 65. The decision was made to take the patient back to the operation room to search for any source of bleeding. It was found that most of the bleeding was coming from the anterior prostate and bladder neck. Additionally, there was some bleeding from the lateral resections and around the verumontanum. Of interest, no arterial or venous sinus bleeding was found. This demonstrates that this bleeding is non-surgical and is, instead, caused by a coagulation abnormality that leads to inability to form clots or protect clots from degradation. A catheter and inflated balloon to 90 cc was placed and pulled down hard to compress any further bleeding. The bleeding was under control. The patient’s bleeding decreased gradually through postoperative day one.

The patient’s coagulation studies following surgery showed a high prothrombin time (PT) level of 19.6 s, a high international normalized ratio (INR) level of 1.7, a normal partial thromboplastin time (PTT) level of 28.2 s, a low fibrinogen level of 176 mg/dL, and a high fibrinogen degradation product (FDP) level of 20 μg/mL. These abnormal labs are consistent with significant fibrinolytic activity that happened after surgery. This can be induced by the effect of urine urokinase and tissue plasminogen activator (tPA) that leaked into the blood stream after TURP surgery. Urokinase may have both local and systemic fibrinolytic effects that may affect blood clot formation or stability [10,11]. Coagulation studies returned to normal gradually after day one.

After surgery, the patient’s labs showed pH of 7.08, bicarbonate of 8 mEq/L, anion gap of 15 mEq/L, and lactic acid of 9.6 mg/dL that are consistent with severe anion gap metabolic acidosis secondary to lactic acidosis type A. Patient did also have hypotension with leukocytosis of 40.5 concerning for septic shock. Subsequently, blood cultures were collected and empiric intravenous antibiotics and levophed drip were started. The patient was then transferred to the intensive care unit (ICU). Of interest, blood cultures came back normal, indicating that this leukocytosis was reactive to body stress after surgery and hypotension. Moreover, the patient developed hypocalcemia. The patient also developed new left bundle branch block on electrocardiogram and high troponin, which are consistent with type II non-ST elevation myocardial infarction (NSTEMI). As expected, the patient’s troponin started trending down and left bundle branch block resolved.

On post operative day one, the patient had clear efflux on slow bladder irrigation. Catheter traction was removed without bleeding or any complications. The patient’s hemoglobin dropped to 6.8, and the patient received two units of packed red blood cells. On postoperative day two, vasopressor support was no longer needed. The patient’s hemoglobin started trending up to 7.8 g/dL. The patient’s PT, PTT, INR, Fibrinogen, and FDP returned to normal limits. The patient was transferred out of the ICU.

## 3. Discussion

In our case report, the patient developed post-operative TURP syndrome, severe acute hyponatremia, non-surgical bleeding, and fibrinolytic changes on coagulation studies. It is known that a post-TURP hemorrhage can be surgical or non-surgical, due to coagulation abnormalities that may prevent blood clot formation or stability. In our case, the patient’s bleeding was found to be coming from the anterior prostate and bladder neck without venous sinus or arterial bleeding. This demonstrates that this post-TURP bleeding is non-surgical, and is likely due to the postoperative fibrinolytic changes that affected clot formation.

Human plasma contains a fibrinolytic enzyme called plasmin and its precursor plasminogen. Urinary fibrinolytic activity in human urine was reported in 1947. This urinary fibrinolytic activity was explained by the presence of fibrinolytic enzymes in human urine [16,17]. Urokinase was found in both urine and seminal plasma in significant concentrations. Urokinase was successfully extracted from human urine [10,11,18,19,20]. Urokinase and its precursor are glycoproteins synthesized by renal, endothelial, and malignant cells [21]. Urokinase leakage from urine to blood may explain the fibrinolytic changes found in our patient postoperatively. These fibrinolytic changes might contribute to the non-surgical bleeding after TURP. Additionally, these fibrinolytic changes are not severe. This may be due to the dilutional effect of the irrigation fluid used during the surgery which decreased the concentration of urokinase and tPA.

Urokinase has been used as a thrombolytic to treat both ischemic stroke and ST elevation myocardial infarction (STEMI) because of its low cost [22,23,24,25,26]. In low-income countries where the health system cannot afford alteplase, urokinase is used as an alternative fibrinolytic [27]. Compared to alteplase, Zhang et al. found that low-dose urokinase has similar outcomes in treating acute ischemic stroke with a higher risk of extracranial bleeding [23]. In another comparative study by Sun at al., they found similar results [27]. However, Bao et al. found that urokinase has a lower risk of bleeding compared to alteplase [24]. In a meta-analysis by Kharel et al., they found that there is no significant difference between intravenous urokinase and alteplase in efficacy or safety; subsequently, urokinase can be a suitable alternative for intravenous fibrinolysis in low and middle-income countries to treat acute ischemic stroke [22]. Of interest, soluble urokinase receptor is used as a biomarker for the prognosis of stroke and STEMI patients [28,29].

Theoretically, after prostate surgery, urokinase and tPA levels can be high in blood and urine due to leakage of urokinase. Subsequently, urokinase may cause fibrinolytic changes and increase the risk of non-surgical bleeding by degrading fibrin clots. As a result, urokinase may increase D-dimer and FDP levels. It may also consume fibrinogen, platelets, and coagulation factors leading to disseminated intravascular coagulation (DIC). Neilsen et al. found that tPA in urine contributes to the postoperative blood loss in patients with BPH after undergoing TURP surgery [30]. They found a significant correlation between tPA in urine and FDP in urine and a significant correlation between the amount of resected tissue and FDP in urine [30]. This means that prostate surgeries increase levels of tissue plasminogen activator locally leading to fibrin clot degradation and increasing the bleeding risk.

**Figure 1:**
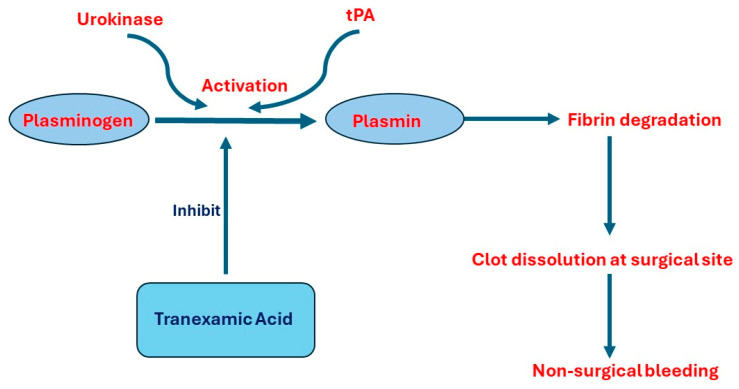
The pathophysiology of developing non-surgical bleeding after TURP and the role of Tranexamic acid. Abbreviations: tPA, tissue plasminogen activation.

Several studies report increased risk of bleeding and changes in the levels of D-dimer, FDP, fibrinogen, platelets, and coagulation factors in patients with BPH after TURP surgery [12,30,31,32]. High levels of tPA and urokinase in prostate cells and urine stream explain these findings. As a result, many clinical trials discussed using an antifibrinolytic agent, Tranexamic acid, locally by injecting it into the bladder and using it in the irrigation fluid during TURP or systemically by injecting it into blood stream during or before TURP [32,33,34,35]. Figure 1 demonstrates the pathophysiology of non-surgical bleeding and the role of Tranexamic acid as a prophylactic agent.

Regarding the use of Tranexamic acid locally, the Tawfick et al. randomized controlled trial found that Tranexamic acid injection into the bladder and using it in the irrigation fluid during TURP reduced post-TURP bleeding without increasing thrombosis risk [32]. They suggested that this bleeding risk is due to tPA release from urothelial cells and urokinase release by the prostate; subsequently, Tranexamic acid injection is theoretically effective to reverse the effect of urine fibrinolytic agents [32]. Although this clinical trial is the first study discussing using Tranexamic acid in the irrigation fluid during TURP, there are other studies that discussed using Tranexamic acid locally in other urological surgeries to decrease post-surgery bleeding [32]. Pourfakhr et al. found that using Tranexamic acid locally during open prostatectomy reduces post-surgery bleeding and prevents hemoglobin drop [36]. In another clinical trial by Bansal and Arora, administration of Tranexamic acid locally in the irrigation fluid was found to reduce perioperative bleeding and the need for blood transfusion in percutaneous nephrolithotomy [37].

On the other hand, many clinical trials discussed using Tranexamic acid systemically by injecting it before or during TURP surgery. The results of these clinical trials support the hypothesis that urokinase and tPA-induced fibrinolysis may cause TURP-induced fibrinolytic changes and increase the risk of post-TURP bleeding [33,34,38,39,40,41,42]. A systematic review and meta-analysis of these clinical trials published in 2022 shows that administration of an anti-fibrinolytic medication, Tranexamic acid, in patients with BPH undergoing TURP reduces blood loss and the drop in hemoglobin levels [35]. A recent 2023 clinical trial that was not included in this systematic review shows similar outcomes. Vanderbruggen et al. found that administration of Tranexamic acid during and after TURP surgery reduces post-TURP blood loss and hemoglobin drop without increasing the risk of thrombosis [43]. This prophylactic effect of the anti-fibrinolytic medication demonstrates the role of urine fibrinolytic enzymes in inducing non-surgical bleeding after TURP surgery. Clinical trials are encouraged to evaluate the safety of Tranexamic acid administration and to search for the appropriate treatment to stop active bleeding after TURP or prevent its occurrence. Of interest, administration of Tranexamic acid systemically was found to reduce post-surgery bleeding and hemoglobin drop after open prostatectomy. It was also found to reduce hospitalization time [44]. Table 1 summarizes all of the clinical trials discussed using Tranexamic acid as a prophylactic agent locally in the irrigation fluid or systemically before, during, or after TURP.

These fibrinolytic changes after TURP are also found in patients with prostate cancer, even without undergoing surgery. This can be explained by the high concentration of urokinase secreted by malignant prostate cells. Moreover, prostate cells are found to express high levels of urokinase receptor in patients with prostate cancer [10,16,17,20,45]. A case report by Wada et al. suggests that high urokinase levels in prostate cancer cells can induce fibrinolytic changes in blood and induce bleeding risk, even in patients without prostate surgery. They suggest treating DIC fibrinolytic changes in patients with prostate cancer with high-dose anti-androgen therapy with combined Tranexamic acid and low molecular weight heparin (LMWH) [12]. This demonstrates the effect of fibrinolytic enzymes in urine and prostate cells and the importance of considering their effect on treating post-TURP hemorrhage or prostate cancer-induced DIC.

The other complication that developed in our case is TURP syndrome. TURP syndrome results from the leakage of irrigation fluid into the intravascular space through the prostatic venous plexus after prostate resection with an incidence of 10% to 15% after TURP. This leakage of irrigation fluid into blood stream can lead to severe hyponatremia and hypervolemia [13,14,46]. Irrigation fluid is used to wash blood and resected tissue away from the surgical site and to distend the bladder. Glycine, mannitol, normal saline, and sorbitol have been used as irrigation fluid during TURP. However, glycine is considered the most commonly used irrigation fluid in therapeutic endoscopic urologic surgeries [47]. TURP syndrome has also been noticed after ureteroscopic procedures and endometrial ablation [13,15,46,48,49,50].

TURP syndrome generally includes hyponatremia, bradycardia, and hypotension that may be preceded by hypertension. In some cases, hypotension can be explained by the hyponatremia and vasovagal reflex due to bladder distension by irrigation fluid [51,52,53]. Hyponatremia can be asymptomatic or present with nausea, vomiting, electrocardiographic changes, convulsions, altered mental status, pulmonary edema, cerebral edema, cardiovascular compromise, and death [54]. This demonstrates the importance of the rapid diagnosis of TURP syndrome. Interestingly, spinal anesthesia is considered the preferred anesthetic technique during TURP to allow early detection of neurologic symptoms indicating acute hyponatremia [55]. TURP syndrome treatment is based mainly on making the patient hemodynamically stable and correcting electrolyte abnormalities [54,55]. In our case, hyponatremia was treated with hypertonic saline followed by normal saline, while hypotension was treated by ephedrine administration followed by levophed drip to keep patient’s mean arterial pressure above 65. This demonstrates the importance of checking patients’ electrolytes after TURP surgery because of the risk of developing severe acute hyponatremia and TURP syndrome.

## 4. Conclusions

Non-surgical bleeding after TURP surgery can be explained by the presence of fibrinolytic agents in the urine, including urokinase released by prostate cells and tPA released by urothelial cells. Tranexamic acid administration locally in the irrigation fluid or systemically in patients undergoing TURP reduces the risk of blood loss and drop in hemoglobin levels. There is a deficiency in studies explaining the cause of fibrinolytic changes and bleeding risk after TURP. More research effort should be directed to explore this topic to understand the pathophysiology of non-surgical bleeding after TURP and to investigate the appropriate medications to treat it. We suggest monitoring DIC profile, including PT, PTT, INR, fibrinogen, and FDP routinely after TURP surgery. We also suggest monitoring patient’s electrolytes and hemodynamic stability after TURP because of the risk of developing severe acute hyponatremia, shock, and TURP syndrome.

## Figures and Tables

**Table 1:** A summary of clinical trials discussing the prophylactic role of Tranexamic acid in preventing post-TURP bleeding.

Reference	Authors	Year	Dose of Tranexamic Acid	Method of Injection	Time of Injection
[42]	Kumsar et al.	2011	10 mg/kg	Intravenous	Operative
[40]	Rannikko et al.	2004	2 g	Oral	Operative and first postoperative day
[39]	Jendoubi et al.	2017	10 mg/kg	Intravenous	Preoperative, intraoperative
[41]	Meng et al.	2018	1 g	Intravenous	Preoperative
[33]	Karkhanei et al.	2020	500 mg	Intravenous	Preoperative
[38]	Gupta et al.	2021	500 mg	Intravenous	Preoperative
[34]	Samir et al.	2022	50 mg/kg	Intravenous	Preoperative
[32]	Tawfick et al.	2022	0.1% TXA 1000 mg (10 mL) in 1 L of irrigation solution during surgery, 500 mg of TXA (5 mL) in 100 mL of normal saline solution at the end of surgery	In the irrigation fluid during the surgery, injection locally into the bladder at the end of surgery	Operative
[43]	Vanderbruggen et al.	2023	loading dose of 10 mg/kg, followed by a maintenance dose of 5 mg/kg/h	Intravenous	Operative, postoperative

TXA, Tranexamic acid.

## Data Availability

The original contributions presented in the study are included in the article, further inquiries can be directed to the corresponding author.
